# Fatigue, stigma, and mood in patients with multiple sclerosis: effectiveness of guided imagery

**DOI:** 10.1186/s12883-022-02677-3

**Published:** 2022-04-22

**Authors:** Mina Beitollahi, Mansooreh Azizzadeh Forouzi, Batool Tirgari, Yunes Jahani

**Affiliations:** 1 Department of Medical Surgical Nursing, Razi Faculty of Nursing and Midwifery, Kerman University of Medical Sciences, Kerman, Iran; 2 Nursing Research Center, Kerman University of Medical Sciences, Kerman, Iran; 3 Modelling in Health Research Center, Institute for Futures Studies in Health, Kerman University of Medical Sciences, Kerman, Iran

**Keywords:** Multiple sclerosis, Guided imagery, Fatigue, Stigma, Mood

## Abstract

**Background and objectives:**

The present study aimed to assess the effectiveness of guided imagery on fatigue, stigma, and mood in patients with multiple sclerosis.

**Methods:**

This clinical trial is a double-blind study that was conducted on 60 patients with multiple sclerosis referred to the largest center for special diseases in the southeast of Iran in 2020. The convenience sampling method was used to select the participants who were later divided into two groups of intervention (*n* = 30) and control (*n* = 30) using block randomization method. The intervention group listened to the guided imagery audio file at home for 25 min. The control group did not receive any intervention. Data were collected by demographic information questionnaires, Fatigue Severity Scale (FSS), Reece Stigma Scale for Multiple Sclerosis (RSS-MS), and the Profile of Mood States (POMS) before and one month after the intervention.

**Results:**

According to the results, there was no significant difference between the two groups before the intervention in terms of the score of fatigue (*P* < 0.0 = 67), stigma (*P* < 0.64), and mood (*P* < 0.17). However, after the intervention, a significant differences was observed in this regard (*P* < 0.0001). In the intervention group, the mean score of fatigue decreased from 59.72 ± 18.32 to 35.8 ± 16.15, and the mean score of stigma decreased from 17.31 ± 15.62 to 5.09 ± 8.06, showing a significant reduction in the levels of fatigue (*P* < 0.0001) and stigma (*P* < 0.0001) compared to before intervention. Also, the mean score of mood decreased from 36.90 ± 12.21 to 28.55 ± 11.87, indicating an improvement in the mood of samples in the intervention group (*P* < 0.0001).

**Conclusions:**

The results indicated that guided imagery, as a cost-effective method, can decrease the fatigue and stigma, and enhance the mood of patients with MS. Therefore, nursing staff can use this method to improve MS patients’ mood and decrease their fatigue and stigma.

## Introduction

Multiple sclerosis is demyelinating, progressive, and inflammatory disease of the central nervous system (CNS) and the most common debilitating disease among young [1] and middle-aged people [2]. On average, age of patients at the onset of MS ranges between 20 and 50 years [3]. The prevalence of MS is 6–200 per 100,000 in the US and currently, around 400,000 people have MS. Iran is considered to have an average level of prevalence, and 50,000 Iranian people are affected by this disease [1]. The prevalence of MS in women is 2–3 times higher than in men [3]. Clinical progression of this disease negatively affects some aspects of life and psychological-behavioral function, and also leads to permanent, debilitation and unpredictable complications [1]. Complications of this disease include emotional and cognitive impairment, weakness, pain, depression, fatigue [4], bowel-bladder dysfunction, sexual dysfunction[3, 4], balance problem [1, 4], social stigma [1], and mood disorder [3]. Fatigue is the most common and earliest symptom of MS and affects 80% of affected people. Fatigue negatively affects people’s function, daily activities, occupation, social activities, and quality of life [5]. Despite the use of pharmacological interventions for moderating the fatigue, side effects of the drugs and failure of these approaches have led to an increasing tendency toward complementary and alternative medicine (CAM) among MS patients [6]. Another complication of MS is stigma [1, 7], which was reported first by Goffman (1963), who defines stigma as a factor that makes people ignore or judge MS patients [1]. Moreover, stigma represents these patients as insignificant people in the eyes of others [8]. Approximately 72% of MS patients suffer from stigma [1]. Stigma has internal and external aspects; the external aspect relates to the negative and reprehensive views as well as unfair and discriminatory behaviors of others. The internal aspect refers to a feeling of being different from others that distorts patients’ self-image, depression, and concealment of the disease [1, 7, 9]. Stigma and its consequences affect peoples’ quality of life and leads to social isolation, loss of job and educational opportunities, unemployment, loss of self-confidence, depression, anxiety, and deprivation of social services [1, 3, 7, 10].

Mood disorder is another symptom observed in MS patients. Mood disorder is defined as a change in people’s attitudes that can lead to a change in social identity that negative effects psychological and mental health. The prevalence of mood disorder is high in MS patients, and it significantly affects their quality of life [8].

Due to the unpredictability of MS, there is no definitive cure for this disease [1], however, there are various methods that can reduce and moderate its symptoms [3]. Literature review indicates that non-pharmacological interventions such as physical, psychological, and cognitive interventions are effective in improving the symptoms of MS [11–16]. Physical approaches include aerobic exercises, resistance training, electromagnetic field therapy, and cooling therapy. Psychological approaches include cognitive behavioral therapy, relaxation therapy, psychotherapy, energy conservation training, progressive muscle relaxation, and educational counseling [5]. The WHO’s recommendation regarding complementary medicine is that people have a right to benefit from the least expensive, safest, and most effective medical interventions for the treatment of their diseases. Currently, in most developed countries, such as USA, England, France, and Germany, complementary medicine methods are being used along with other medical treatments [17]. The prevalence of using at least one of the complementary medicine treatments in one year has been reported at 56% and 52.5% in European countries and in Iran’s capital, respectively [18].

Guided imagery is one of the methods of complementary medicine [2], which was used in the present study. Guided imagery is defined as the generation or recall of different mental images, such as perception of objects or events, and can engage mechanisms used in cognition, memory, and emotional and motor control. Guided imagery is the use of mental visualization (mental images) to improve mood and physical well-being. A mental image can be defined as “a thought with sensory qualities.” It is something we mentally see, hear, taste, smell, touch, or feel [2, 6, 19]. Guided imagery methods affect the hypothalamic–pituitary–adrenal axis and uses human senses and based on the concept that the mind and body are connected, affects the body and psyche [20].Considering the potential effects of immune system mediators, guided imagery uses guided images to treat immune system disorders such as MS by causing a change in the hypothalamic–pituitary–adrenal axis [2]. This medical intervention has many advantages; for instance, it improves quality of life, physical function, fatigue, pain, anxiety, depression, daily activities, and adherence to drug regimen [5].

Different studies such as the study of Kaur et al. (2019) have shown that mental imagery is an innovative, effective, cost-effective, and convenient method that can be used as an independent or adjuvant method for treating neuropathic pain in MS patients, MI, stroke, and spinal cord injury [21]. Moreover, the study of Case et al. (2018) revealed that Healing Light guided imagery, compared with a wait-list control has positive effects on mood, fatigue, and quality of life in patients with relapsing–remitting multiple sclerosis [22]. They did not study all types of MS and also, they did not follow up on patients. Arseny et al. (2018) reported that people’s ability to prevent, reduce, and ameliorate cognitive impairments has improved in MS patients by the advances made in pharmacological, neurological, psychological, and physical medicines, but there are still some deficiencies [23]. Chamanzari et al. (2013) conducted a study to assess the effects of guided imagery on the pain of orthopedic surgeries among traumatic patients referred to Hashemi Nezhad Hospital in Mashhad and their results indicated that guided imagery reduced pain and duration of pain, and also improved pain quality on the third day after the surgery in patients with fracture, while it did not affect pain intensity [24]. Jessica et al. (2020) conducted a study to compare the effect of hypnoanalysis and guided imagery on the disability, quality of life and number of relapses of MS patients. Their results showed that, compared to hypnoanalysis, guided imagery intervention improved two subscales of quality of life in MS patients [25]. They did not assess fatigue, stigma and mood. Therefore, there is a need for more studies to determine the effects of guided imagery on fatigue, stigma, and mood in MS patients.

Employing complementary medicine in nursing is not new, and has been one of the interests of nursing theoreticians for a long time [26, 27]. However, nowadays, more attention is being paid to complementary medicine in the nursing profession due to changes in people’s preferences. Ethical commitments, acting as strong stimuli, drive nurses toward using complementary medicine to moderate the problems of patients [28]. This study is aiming at assessing the effect of guided imagery on reduce fatigue and stigma, and also improve mood in MS patients in Iran.

### Purpose

Determining the effect of guided imagery on fatigue, stigma and mood in MS patients.

### Hypothesis

1. Guided imagery has an effect on the fatigue of MS patients.
2. Guided imagery has an effect on the stigma of MS patients.
3. Guided imagery has an effect on the mood of MS patients.

## Methods

### Design

The present study is a double-blind clinical trial that was conducted on MS patients who referred to the Medical Center of Special Diseases, which is the largest center in the southeast of Iran. Due to the intervention was performed at home, statistics Specialist and participants did not know which data are related to intervention or control group, and this showed that the study was double-blinded.

### Sample

The sample size was calculated to be 70 patients (*n* = 35 in each group), using the sample size formula and standard deviation extracted from the study of Case et al. (2018) with 90% power. Samples were selected by convenience method and based on the inclusion criteria. Using block randomization method with block size of 4, and also R software, two participants were placed in each of the intervention and control groups within each block, and then, patients were randomly divided into control (35) and intervention (35) groups.

## Inclusion criteria

Being 18–70 years old, to sign a written informed consent, having full consciousness, having auditory, visual, and verbal abilities, being able to read and write in Farsi, being diagnosed with relapsing–remitting MS by the neurologist, suffering from fatigue, and obtaining the score of less than 6 based on the FSS questionnaire were among the inclusion criteria.

## Exclusion criteria

Using sedative, Antidepressant or opioids, being absent for more than two sessions of the intervention, having a history of mental disease and continuous hospitalization, suffering from other nervous disorders, and having the experience of using relaxation methods (guided imagery).

## Intervention

Patients were selected according to the inclusion criteria. The patients in the control group did not receive any intervention. However, patients in the intervention group in addition to their standard medication, listened to the guided imagery audio files for 20 min every day expect for Friday, for 12 sessions a week. The researcher held a briefing session for the intervention group and gave them the audio file. All the necessary explanations regarding listening to the guided imagery audio file were given to the patients. To reduce noises, after talking to patients and their families, a room was dedicated to the patients for 20 min to listen to the audio file. The patients listened to the audio file every week for 4 weeks in the morning (between 8:00 and 9:00) and evening (between 20:00 and 21:00) according to previous studies [29]. The transcript of the audio file included expressions for imagining desirable sceneries (forests, beaches, holy places…) along with proper effects and positive emphasis on reducing fatigue and stigma, and improving mood. The transcript was designed by the researcher based on Farsi and English sources and under the supervision of experts, which and finally was approved by the audio center of Kerman University of Medical Sciences. Patients were allowed to receive standard MS medication in both groups. During the study, in addition to sending reminder short messages, the participants were reminded to listen to the audio file and their possible questions were answered through phone calls.

## Data collection

1. Demographic questionnaire that included questions about personal information such as age, gender, marital status, education, occupation, family’s monthly income, and tobacco use, and medical information such as disease type (relapsing–remitting MS (RRMS), primary progressive MS (PPMS), secondary progressive MS (SPMS), and progressive-relapsing MS (PRMS)), as well as medication type, disease duration, number of relapses in the past year, and having other family members with MS.

2. The FSS questionnaire was used to assess the level of fatigue. This tool was designed by Krupp et al. (1989) and consists of 9 questions on 7-point Likert scale, ranging from 7 (the highest level of fatigue) and 1 (the lack of fatigue). To calculate the total score of fatigue, the scores attributed to each of the items were added and the sum was divided by 9, this way the average score was obtained. A lower score indicated lower fatigue [30, 31]. The validity and reliability of this questionnaire have been analyzed and confirmed in various studies. For instance, Farahani et al. (2012) investigated the reliability of the Iranian version of this tool in a sample of MS patients, and reported its internal consistency at 0.96 based on Cronbach’s alpha [32].

3. The RSS-MS questionnaire was used to assess stigma in patients. This tool was specially designed for the assessment of stigma in MS patients by Smith et al. (2019). It consists of 9 questions on 5-point Likert scale, ranging from 1 (never) to 5 (always). To measure the total score of stigma, the scores given to the items are added, and a higher score shows higher level of stigma [18]. The validity of this questionnaire has been confirmed by Smith et al. (2019), and its reliability has been reported to be 0.91 based on Cronbach’s alpha.

4. POMS questionnaire was used to assess mood. This tool was designed by Nirola and Draplin in 1971. The validity and reliability of this tool have been evaluated in different studies such as those conducted by Albert et al. (2009) and Iwamitus et al. (2003). This questionnaire has 65 items and 6 subscales including anxiety/tension (9 items), depression/dejection (15 items), fatigue (7 items), confusion/bewilderment (12 items), anger/hostility (12 items), and ability/vigor (12 items). In this study, seven extra items were added to enrich the questionnaire, and therefore, they were not included in the scoring. The score ranges from zero to four based on the Likert scale. To calculate the total score of mood, the scores of 5 negative factors including anxiety, depression, anger, fatigue, and confusion are summed up and added and sum score of positive factor, which has been obtained by summing up the score of negative factors. Therefore, the total score of mood ranges from 0 to 168, with the lower score indicating higher mood [33]. In Iran, this tool has been used by Tirgari et al. (2006) who reported an acceptable validity for it. They also reported its reliability to be 0.81 based on Cronbach’s alpha.

## Statistics

The collected data were entered into SPSS software version 22. Using dispersion and central indicators, independent sample t-test, paired t-test, Chi-Square, Fisher, and analysis of covariance (ANCOVA), the data were analyzed at the significant level of 0.05.

## Ethics

The present research was conducted after obtaining the code of ethics (IR.KMU.REC. 1398.263) from the Ethics Committee of Kerman University of Medical Sciences and Clinical trial code: IRCT20151107024919N11 from Iranian Clinical Trial Center. After obtaining a written consent from patients, the first researcher explained the objective and method of study to MS patients, and emphasized on the principles of confidentiality and anonymity. The researcher also explained that the participation in the study is voluntarily, so participants can freely withdraw from the study at any time. In order to preserve the ethical issues, the audio file was provided to the patients in the control group at the end of the study, and also the results of the study were reported to the authorities and participants.

## Results

Among the 70 MS patients in this study, the data of 60 patients were analyzed. Finally, data analysis was performed on 60 participants (30 participants in the intervention group and 30 in the control group). 5 participants in the intervention group (2 women due to hospitalization for COVID-19 and 3 women due to not doing the assignments of more than two sessions) and 5 participants in the control group (1 woman and 1 man due to unwillingness to continue and 3 women due to family issues) were excluded from the study.

According to the results, the mean age of participants was 39.73 years (± 9.45) and 38.40 years (± 10.29) in the intervention and control group, respectively, and the t-test did not reveal any significant difference between the two groups in terms of age (*P* > 0.05). Most participants in both groups were female, married, housewives, and had a high school diploma or higher education. There was no significant difference between the two groups in terms of demographic variables (*P* > 0.05), (Table 1).

**Table 1:** Comparison of demographic variables between groups

**Variable**		**Control N) %)**	**Intervention N) %)**	**Statistic**	**p**
**Gender**	Male	4 (13.3)	3 (10)		0.99
	Female	26 (86.7)	27 (90)		
**Marital status**	Single	4 (13.3)	5 (17.6)		0.99
	Married	22 (73.3)	32 (73.3)		
	Other	4(13.3)	3 (10)		
**Education level**	High school	15 (50)	13 (43.3)	*χ*2 = 0.26	0.60
	Abovediploma	15 (50)	17 (56.7)		
**Job**	Employed	13 (43.3)	12 (40)		0.40
	housewife	16 (53.3)	14(46.7)		
	Student	0 (0)	3 (10)		
	Unemployed	1 (3.3)	1 (3.3)		
**Type of disease**	Recurrent and receding	23(76.7)	25(83.3)	*χ*2 = 0.41	0.51
	Progressive recurrent	7(23.3)	5(16.7)		
	Early Progressive				
	Secondary progressive		6(20)		
**monthly income**	One million and less	5(16.7)	17(56.7)	*χ*2 = 0.15	0.92
	Between one and three million	17(57.7)	7(23.3)		
	Between three and five million	8(26.7)			
**Time of onset of the disease**	One to five years	11(36.7)	8(26.7)	*χ*2 = 0.69	0.70
	Five to ten years	11(36.7)	9(30)		
	Ten years and older	8(28.7)	28(93.3)		
**Recurrence in One year ago**	Once or twice	26(86.7)	2(13.3)		0.67
	More than twice	4(6/7)	8(26.7)		
**Type of drug used**	Interferon beta	13(43.3)	4(13.3)		0.21
	Glatiramer acetate	1(3.3)	3(10)		
	Fingolimod	5(16.7)	3(10)		
	Rituxymab	1(3.3)	8(26.7)		
	Diphosel	5(16.7)	0(0)		
	Other	3(10)	4(13.3)		
	None	2(6.7)	0(0)		
**drug use**	Yes	0(0)	30(100)		
	No	30(100)	4(13.3)		
**Family history**	Yes	9(30)	26(86.7)	*χ*2 = 2.45	0.21
	No	21(70)			
**Age**		M±SD (39.73±9.45)	M±SD (38.40±10.29)	t=0.52	0.6

1. Fatigue: According to the results of paired t test, when the patients' pre and post guided imagery intervention mean scores for fatigue were evaluated, it was determined that patient's mean score for fatigue had significantly decreased in the intervention group (*P* < 0.0001); while, in the control group there was no significant difference in patients' pre and post intervention mean scores of fatigue (*P* > 0.05), (Table 2).

**Table 2:** Comparison of fatigue, stigma and mood in each group before and one month after Intervention

Variable	Time	Control group				Intervention group			
		Mean	SD*	T*	p	Mean	SD	T	p
**Fatigue**	Pre intervention	57.97	13.33	7.32	0.99	59.72	18.32	7.89	*P* < 0.0001
	One month after intervention	57.97	12.36			35.81	16.15		
**Stigma**	Pre intervention	19.62	22.57	1.11	0.27	17.31	15.62	5.39	*P* < 0.0001
	One month after intervention	16.85	20.96			9.53	9.75		
Mood	Pre intervention	40.59	8.09	0.49	0.62	36.90	12.21	4.75	*P* < 0.0001
	One month after intervention	39.88	6.95			28.55	11.87		

* *SD* Std.Deviation, *T*Paired.Samples T Test

The mean score of fatigue before the implementation of guided imagery intervention was 57.97 (± 13.33) and 59.72 (± 18.32) in the control and intervention groups, respectively. Accordingly, there was no significant difference in the fatigue score between the two groups before the intervention (*P* > 0.05). However, the mean score of fatigue after the guided imagery intervention decreased by 23.64 in the intervention group. Therefore, it can be concluded that the level of fatigue was significantly different after the intervention (*P* < 0.0001), (Table 3).

**Table 3:** Comparison of fatigue, stigma and mood between two groups before and one month after intervention

Variable	Time	Control group	Intervention group	Statistics	p
		Mean (SD*)	Mean (SD)		
**Fatigue**	Pre intervention	57.97(13.33)	59.72(18.32)	T* = 0.42	0.67
	One month after intervention	58.42(5.68)	34.78(5.68)	F* = 47.27	*P* < 0.0001
**Stigma**	Pre intervention	19.62(22.57)	17.31(15.62)	T = 0.46	0.64
	One month after intervention	16.05(1.73)	10.33(1.73)	F = 5.43	0.023
Mood	Pre intervention	41.21(12.58)	37.39(8.28)	T = 1.38	0.17
	One month after intervention	29.63(3.29)	38.79(3.29)	F = 20.65	*P* < 0.0001

* SD: Std.Deviation, *F: ANCOVA, *T: Independent.Samples T Test

2- Stigma: According to the results of paired t test, when the patients' pre and post guided imagery intervention mean scores for stigma were evaluated, it was determined that patient's mean score for stigma had significantly decreased in the intervention group (*P* < 0.0001); while, in the control group there was no significant difference in patients' pre and post intervention mean scores of stigma (*P* > 0.05), (Table 2).

The mean stigma score before the guided imagery was 19.62 (± 22.57) and 17.31 (± 15.62) in the control and intervention groups, respectively. Accordingly, the scores of stigma were not significantly different between the two groups (*P* > 0.05). However, the mean score of stigma decreased by 5.72 in the intervention group, so that the level of stigma was significantly different after the intervention (*P* < 0.0001), (Table 3).

3- Mood: According to the results of paired t test, when the patients' pre and post guided imagery intervention mean scores for mood were evaluated, it was determined that patient's mean score for mood had significantly decreased in the intervention group (*P* < 0.0001); while, in the control group there was no significant difference in patients' pre and post intervention mean scores of mood (*P* > 0.05), (Table 2).

The mean score of mood before the guided imagery was 41.21 (± 12.58) and 37.39 (± 8.28) in the control and intervention groups, respectively, so there was no significant different between the two groups in terms of the score of mood(*P* > 0.05). However, the mean score of mood increased by 9.16 in the intervention group after the guided imagery intervention. As a result, the level of mood was significantly different after the intervention (*P* < 0.0001), (Table 3).

## Discussion

The results showed that guided imagery can significantly reduce fatigue and stigma, and also improve mood in MS patients. These results are consistent with the results of two studies that investigated the effect of guided imagery in MS Patients. Case et al. (2018) assessed the effects of healing light guided imagery compared with a wait-list control in patients with relapsing–remitting multiple sclerosis. Their results showed that, healing light guided imagery is effective in reducing patients’ fatigue and improving their mood and quality of life [22]. They did not include all types of MS and also, they did not follow up on patients. In the present study, we included all type of MS and our results showed auditory guided imagery is effective on fatigue, stigma and mood of all type of disease at one month after intervention.

Jessica et al. (2020) compare the effect of hypnoanalysis and guided imagery in MS patients. Outcome measures, disability (the Expanded Disability Status Scale, EDSS), quality of life (QoL, measured with the SF-36) and number of relapses, were evaluated. They showed that, compared to hypnoanalysis, guided imagery improved quality of life in MS patients. They did not assess fatigue. Fatigue in MS patients occurs due to damage done to the hypothalamus adrenal axis. So probably, the effects of guided imagery are exerted through relaxation and activation of damaged parts in the brain [22].

Some studies have used guided imagery to reduce fatigue in different diseases. Beizaee et al. (2017) examined the effects of guided imagery on hemodialysis patients, and showed that the intervention led to a decrease in patients’ fatigue [34]. Buyukbayram et al. (2021) reported that guided imagery had a positive effect on cancer patients’ fatigue [35]. The results of another study conducted by Lee et al. (2013) indicated that guided imagery led to a decrease in cancer patients’ fatigue [36]. Although medical diagnoses in above studies have been different from that of the present study, these studies included chronic diseases, and all chronic diseases have some common features such as long-term complications of treatment, long-term use of medication and their side effects, frequent and lengthy hospitalization, fear of future, and sleeplessness, and patients are forced to live with the disease.

According to the results of the present study guided imagery reduced stigma in MS patients. The literature review did not reveal any studies investigating the effect of guided imagery on stigma in MS patients. Since guided imagery affects people’s thoughts and imagination, it has been able to affect people’s minds and repel the feeling of stigma through imagery [37]. Kolayiş (2015) stated that guided imagery, in addition to improving the physical health, improves peoples’ mental status; for instance, by controlling emotions and focus [38].

Our results showed guided imagery improve mood in MS patients. Case et al. (2018) reported that guided imagery improved MS patients’ mood [22]. In guided imagery, patients are asked to imagine desirable images and sceneries such as flowers, plants, and sea, and use regular breathing exercises, which distance the mind from problems and create peace in patients, thereby improving their mood. Tsitsi et al. (2017) reported that relaxation intervention (progressive muscle relaxation and guided imagery techniques) had positive effects on anxiety and improved the mood of parents of hospitalized children with malignancies [39]. Shahabi et al. (2020) stated that in guided imagery, people are encouraged to take deep abdominal and diaphragmatic breaths and then release their muscles and imagine sceneries such as forests, beaches, and holy places, and pay attention to the smells and sounds around them. Studies have shown that focusing on positive visualization and imagery can relax and balance the mood. In fact, guided imagery increases resilience through distracting the mind from disturbing stimuli, relaxing patients, and affecting patients’ mood [40].

## Strengths and limitations

The strengths of present study include; having more training sessions, having follow-up one month after the intervention, and being double-blind trial. On the other hand, weaknesses of this study include; lack of possibility of holding in-person and shared sessions due to COVID-19, which eliminated the synergic effect of shared sessions and the possibility of monitoring the intervention group, and the concurrence of sampling and COVID-19 pandemic**.**

Limitations of this study include; lack of possibility of monitoring the intervention at people’s homes, modest sample size, the concurrence of sampling and COVID-19 pandemic, and use of convenience sampling. Therefore, it is recommended that in future studies on this issue, larger sample sizes should be used.

## Conclusion

The results of present study indicated that guided imagery, as a non-invasive method of complementary medicine, has positive effects on reducing fatigue and stigma, and also improving the mood.

Guided imagery, as a non-invasive method of complementary medicine, can reduce the complications of MS. It is recommended that nursing managers improve the nursing staff’s knowledge of no-invasive methods such as complementary medicine by arranging in-service training courses and informing them about the significance of these techniques in improving MS patients’ fatigue, mood, and stigma. Guided imagery is a useful and cost-effective method that does not need special tools and moreover, does not cause any complications and is implementable in all situations. Therefore, the results of this study, in addition to being useful in nursing care, can be a basis for future studies.

## Data Availability

The datasets used and/or analyzed during the current study are available and the corresponding author can deposit the data.
